# Testing Off-the-Shelf Optical Wireless LANs for Smart City Environments

**DOI:** 10.3390/s21165451

**Published:** 2021-08-12

**Authors:** Loreto Pescosolido, Emilio Ancillotti, Andrea Passarella

**Affiliations:** IIT-CNR-Institute for Informatics and Telematics-Italian National Research Council, 56124 Pisa, Italy; emilio.ancillotti@iit.cnr.it (E.A.); a.passarella@iit.cnr.it (A.P.)

**Keywords:** LiFi, IR/VLC, optical wireless LAN, smart cities, smart home

## Abstract

Optical wireless LANs (OWLs) constitute an emerging networking paradigm for indoor scenarios’ fit to different smart cities’ fields of applications. Commercial products employing this technology have been made available on the market in recent years. In this work, we investigate, through a set of indoor communication experiments based on commercially available products, how different environmental and usage modes affect the performance of the system, addressing the presence of multiple users, the position and mobility of the mobile devices, the handover among adjacent cells and the effect of background lighting. Our finding shows that the system is quite robust with respect to the variation of operational conditions. We show that, in most conditions, the links can reliably sustain a stable throughput, achieving at least 50% of the throughput achieved with using the maximum light intensity of the transmitting lamp, while they are affected in a very mild way by factors like position and height of the mobile device, and virtually unaffected by variations in the background light.

## 1. Introduction

In future smart cities, the variety and amount of IoT devices will be much richer and much more connected with respect to most common nowadays scenarios. In both outdoor and indoor environments, traditional human-operated devices such as smartphones, tablets and laptops, are constituting an ever decreasing share of the overall amount connected devices [1].

For indoor environments, energetically sustainable smart home and smart building appliances [2], ambient assisted living, health and lifestyle monitoring and assistance through different sensors and biomedical devices, and home automation appliances [3], are expected to contribute to an increase in the number and variety of IoT devices in use. Similar forecasts hold for offices, hospitals, and industrial environments [3]. IoT devices can be used to interact with the user and collect data not only for application limited to the users’ own scope. They can indeed provide meaningful information to the smart city. Electricity and water consumption monitoring tools are a useful source of data in smart buildings for sustainable smart cities. Monitoring the users’ behavior indoor (home, office, restaurants, etc.) can help predict, for instance, user movements across different areas of the city, and so on. In outdoor environments, and particularly in vehicular scenarios, vehicles are expected to frequently exchange information among themselves and with roadside units in order to optimize the overall efficiency of the transportation system, reduce road traffic and accidents, and coexist with pedestrians in a safer way.

It is well understood that, due to both the increased number of connected devices and the increased number of services and applications, these scenarios will require much more bandwidth than most common ones nowadays. Two technologies that allow to exploit an unprecedentedly large portion of the electromagnetic spectrum, and which have emerged during the last decade by achieving a sufficient degree of maturity to hit the market, are visible light communications (VLC) and infrared (IR) communications. Wireless networks based on these technologies are also known as optical wireless LANs (OWL), or Light Fidelity (LiFi) networks.

The visible light spectrum extends from 430 to 750 Terahertz (corresponding to wavelengths in the range between 400 and 700 nm), whereas the so called near-infrared spectrum (which is typically used by commercial devices) ranges, nominally, from 214 to 400 THz (wavelengths in the range 780 nm e 1 mm). Although research in the area of IR/VLC communications has, as of today, a 40-year history, starting from the early 1980s it became apparent that the challenge to manufacture transceivers suitable to exploit this band would have taken some time. The availability of low cost Light emitting diodes (LEDs) at the end of the 1990s triggered a strong impulse to the research in the area. The gain in terms of the raw bit rate achieved by VLC research prototypes during the last decade has been 100-fold, ranging, e.g., from the 100 Mbps available in 2009 [4], through 1 Gbps in 2013 [5], up to 10 Gbps in [6]. In parallel, commercial products started to be available in recent years from several manufacturers. Despite the actual throughputs achieved by currently available off-the-shelf devices is lower than those achieved by cutting edge research works, these systems are already being used in different scenarios, and exhibit quite interesting features.

The two closely-related technologies, IR and VLC, perfectly combine in the design of transceivers able to operate in indoor environments like houses, offices, hospitals, farms, etc. In fact, the illumination lamps inherently present in these environments provide a natural network infrastructure made of access points (APs), that can be connected to the backbone wired section of a LAN. On the other hand, mobile devices (IoT devices, laptops, etc.) can transmit in the IR band, thus avoiding the presence of undesired light sources from places different from the room ceiling or the upper ends of the walls.

The potential impact of IR/VLC networks is expected to be considerable due to their unique characteristics in terms of offered bandwidth, hardware cost, energy consumption and inherently added security, see Section 2. All these aspects make IR/VLC communications and networks a promising technology that will greatly contribute to enabling the smart city paradigm to work in a sustainable, cost efficient, and performing way.

In this work, we consider the use of IR/VLC in indoor environments. More specifically, we evaluate the performance of a IR/VLC LAN built with off-the-shelf commercially available products focusing on key environmental, deployment, and multiple access conditions, and the way they affect performance in both the uplink and downlink direction. This kind of LAN can be deployed as an alternative to, or alongside with, more traditional systems based on well-established wireless technologies such as WiFi, Bluetooth, Bluetooth Low Energy (BLE), Zigbee (or more generally, systems based on standards of the IEEE 802.15.X family, typically used in IoT and parameter acquisition systems).

The key findings of our experiments can be summarized as follows:

- The system gracefully tolerates the presence of multiple transmitting mobile devices, as the aggregate date rate even shows an increase as more and more mobile units (MUs) are added to the picture.
- The downlink performance proves to be reliable with respect to an increase of the distance between the AP transmitter and receiver, and to a decrease of the AP transmitting lamp luminosity, although they do depend on these parameters. In the uplink, the AP lamp luminosity does not affect the performance, provided that a minimum level of luminosity is kept in order to guarantee the ACK (or other types of signalling messages) reception on the MU side. Otherwise, the uplink communication is disrupted.
- Both the downlink and uplink performance are substantially independent of the off-nadir distance of the mobile unit with respect to the AP, as long as the receiver is in the cell coverage, considering that this includes the effect of a roughly 30 degree variation of the angle of orientation of the mobile unit with respect to the AP-MU direction.
- In the considered environment, with no direct sunlight impinging on the receivers, both the downlink and uplink performance are essentially not affected by the background light.

This work is organized as follows: in Section 2 and Section 3 we provide some background information on the applications in indoor smart city environments (Section 2) and on the IR/VLC technology (Section 3). In Section 4 we describe the testbed we used for our measurements. In Section 5 we present and discuss our measurements and results. Finally, Section 6 concludes this study, summarizing our key findings.

## 2. IR/VLC Communications and Networking in Smart Cities Scenarios

Communications exploiting electromagnetic waves in the infrared and visible light spectra have been an active filed of research, as of today, for over 40 years. Both type of emission can be achieved, as of today, with low-cost light emitting diodes (LEDs) and photo diodes (PD) at the receiver. The two different spectra can be considered complimentary due to several characteristics they have in common and other aspects in which they differ. Communications in the IR/VLC spectrum can alternatively be based on narrow “light” beams, which convey the optical power in the Line of Sight (LOS) between transmitter and receiver, or diffuse radiation. The first type of communication allows to save power (as the energy is not dispersed over a wide angle of emission) but is very sensible to the angle of departure (at the transmitter) and angle of arrival (at the receiver). On the other hand, diffuse radiation can allow for wide angles (above 45 degrees), thus being robust to transmitter-receiver pointing angles misalignments. In general, diffuse radiation based systems require a higher power to achieve a target SNR at the receiver, with respect to the beam-based ones. However, multipath reflections may help partially reduce the gap, provided that a suitable modulation technique is in use. In the remainder of this work, we consider systems based on diffuse radiation, which are more suitable to the smart home and other applications fit to smart city scenarios described below.

Typical applications fitting to smart cities scenarios, envisaged for optical wireless communication networks, include at home patient monitoring of biomedical signals, [7,8,9,10], or hospital environments [11,12]. Another interesting application is the detection/classification of the human movement at home [13,14,15], which can also be used for the monitoring of fragile or elderly people behavior. This intrinsic capability can be used also, for instance, to track/guide/assist customers inside shopping malls, museums, etc. [16,17]. Home automation is also another application field where optical communication technologies can be an effective enabler. There are quite a few studies, e.g., where the market potential of the smart home paradigm, along with the related barriers and risks are analyzed [18,19,20,21,22,23] and IR/VLC based indoor networking has been shown to be a viable option in these scenarios [24,25,26,27,28,29,30,31].

There are a number of reasons that make IR/VLC networks very attractive in all these scenarios. The most important are the following:

(i) The already mentioned impressive bandwidth of the visible light and infrared spectrum, which will complement traditional Sub-10 GHz communications as well as mmWave based ones.
(ii) The energetic sustainability of VLC based solutions, which can exploit LED lamps inherently present inside buildings (for illumination purposes) or on the road (traffic lights, lamp posts) as transducers. In other words, these systems reuse, for the communication purpose, the energy already consumed to keep the lamp on for other purposes.
(iii) The competitiveness in terms of costs of the required hardware, which is expected to become comparable, in the next few years (if not already so), with that required by traditional wireless networks, due to the extreme low cost of the essential components of the transceivers: LEDs and photo-diodes.
(iv) The inherent security of the communications, due to the fact that, in an indoor environment, optical signals are quite harder to eavesdrop than more traditional RF signals for eavesdroppers placed outside the indoor environment.

## 3. Background on the IR/VLC Technology Evolution

The research on communication and networking devices on optical transceivers, i.e., transceivers operating in either the visible light or infrared spectrum bands, has been tightly coupled with the availability on the market of low cost LEDs. Hardware cost, the fact that visible light LEDs are currently widely used for illumination purpose, i.e., they are present anyway in both indoor and outdoor scenarios, and bandwidth availability are arguably the most important factor to make this technology competitive with respect to RF-based ones. In the following, we provide a brief overview of the development of optical communication technology, which allows us to put into context the experiments and findings provided in the following sections.

Low cost LEDs for the IR spectrum appeared first, and studies dating back to the late 1970s, e.g., [32], provided a proof of concept, in a mixed simulation and experimental based setup, of transmission being possible at 100 Kbps, with the main limiting factor being the limited modulation bandwidth of the LEDs. In 1994, an aggregate rate of 7.5 Mbps for 5 users (1.5 Mbps per user) was achieved using diffuse IR radiation with PPM-CDMA modulation [33]. Later on, experimental prototypes achieving 6 Mbps [34] and 50 Mbps [35] were presented. In the majority of the most significant studies considering IR communications, multiple access is obtained through CDMA [36,37]. As of today, IR links can be established at tens of Mbps using commercially available products, including the ones we have used for this work; see Section 4.

In the case of visible light, low cost LEDs in the green and blue bands were made commercially available in the late 1990s (while LEDs in the red spectrum had already been available for some time), thus allowing to implement three-led based RGB transmission systems. For such systems, the potential to achieve hundreds of Mbps with a diffuse link (and up to 10 Gbps and beyond with narrow beam links), thanks to the exceptional bandwidth of the visible light spectrum, was highlighted in [38]. Later studies based on low cost single chip white LEDs (i.e., capable of covering the entire visible light spectrum with a single LED, as opposed to the use of three LEDs to obtain red, green, and blue emissions), that were made available on the market in the mid 2000s, presented prototypes achieving tens Mbps [39,40], up to 100 Mbps [4] by 2009. These works deal with one of the major problems of VLC systems, i.e. the need to equalize the LED spectrum response, which has a limited modulation bandwidth. More recent works focused on different multiple access techniques. For the visible light spectrum-based transmissions, with respect to those assuming IR transmissions (with which CDMA often preferred), the most appealing techniques are OFDMA [41,42], and NOMA [43,44,45,46,47]; see [48] for an in-depth survey. Despite there have recently been studies based on low cost prototypes capable to transmit at bit rates in the order of 10 Gbps [6], currently commercially available products based on VLC operate with single user bit rate in the order of several tens Mbps.

The typical indoor deployment of an IR/VLC network includes multiple APs mounted on the ceiling, forming a set of so called “attocells”. The typical coverage radius of these cells is 3–4 m, depending on the ceiling height. Wireless devices, equipped with suitable transceivers, are scattered in the room, either in fixed positions, or mobile. One popular configuration of such systems operates in the visible spectrum for the downlink an in the IR spectrum for the uplink [49]. Accordingly, the APs are equipped with a LED lamp, for transmission, and an IR sensor, for reception. The wireless devices are equipped with a light sensor for receiving signals from the AP, and an IR LED for transmission. This network architecture requires us to manage the association between devices and cells [50] and to cope with user mobility in the handover of users among cells [51].

## 4. Testbed Description

We built a test environment for the performance evaluation of an IR/VLC based indoor wireless LAN capable of supporting high throughput applications.

**Figure 1:**
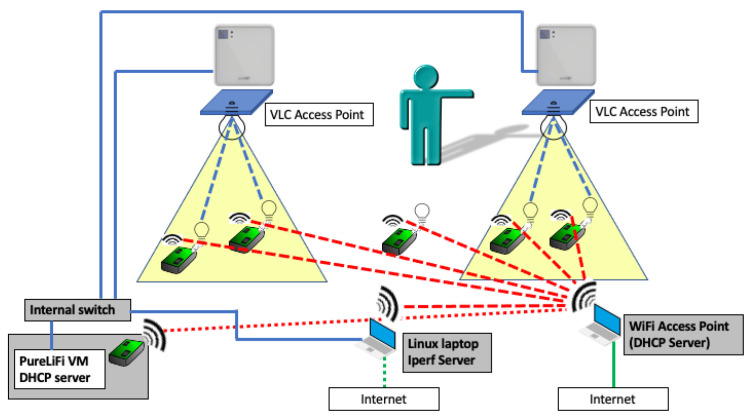
Testbed sketch.

The considered system consists of two APs and six mobile devices that can represent different data sources (sensors) present in the home environment, each equipped with a IR/VLC dongle. The considered scenario, with 2 APs and 6 mobile devices, is sketched out in Figure 1.

**Figure 2:**
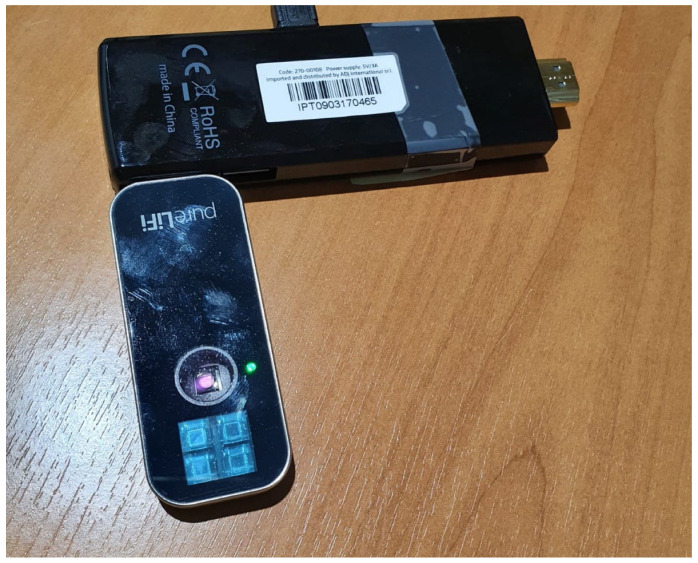
Mobile device.

The mobile devices used in the testbed are of the type shown in Figure 2. Each mobile device is composed of two elements:

- In the upper part of the figure: PC-stick ADJ 270-00108 equipped with Intel Atom Z8350 processor, 2 GB RAM and 32 GB eMMC hard disk, 802.11 a/b/g/n/ac WiFi card, Bluetooth 4.0, 1 USB 2.0 port, 1 USB 3.0 port, 1 HDMI port. Operating system: Linux.
- In the lower part of the figure: IR/VLC PureLifi LiFi-XC Station Dongle, the transmit sensor and the receive sensor are visible.

Of course, the IR/VLC dongles can also be plugged into traditional laptops, for example to support remote medical assistance sessions via video streaming, as the throughput supported both in uplink and downlink is adequate for this type of application.

The mobile devices are controlled through a WiFi network hosted by a conventional WiFi AP running on a Windows laptop (bottom left in Figure 1), which hosts a classic DHCP server. This network is used to remotely access the devices for configuring them. To the same WiFi network, a Linux laptop (bottom center) and an additional PC-stick (bottom left) are connected. The Linux laptop is used to run the performance analysis software tool used in our measurement, called iPerf [52], which is of common use in the literature for network speed tests, and reaches the mobile devices through the WiFi network. The additional PC-stick hosts a virtual machine (VM) provided by the vendor of the LiFi APs and dongles (see below), which controls the LiFi network. The VM is configured to see the PC-stick network interface (a USB-to-LAN converter) and uses it to reach the two LiFi APs through standard LAN cables (solid blue lines) and a cable switch, to which the Linux laptop is also connected. The Linux laptop is also used as a user interface to control the VM running on the additional PC-stick. The reasons to have the WiFi DHCP server, the iPerf software, and the VM running the LiFI DHCP server on separate machines are (i) to obtain two completely separated IP spaces, also separated by the office intranet IP space, and (ii) to have a dedicated machine for the iPerf tool, which is a heavily computational intensive and could impair the availability and functioning of the DHCP servers.

The LiFi APs (of the PureLifi LiFi-XC AP model) and dongles are produced by PureLifi [53], a company raised as an academic spin-off in the early 2010s, and one of the first to enter the new market of IR/VLC-based wireless networks. In future works, we will include other vendors’ equipment to provide a more complete picture. Still, our results in terms of reliability (see Section 5) of the IR/VLC technology show that it has the potential to provide a reliable connectivity under many environmental and deployment conditions.

Each LiFi AP is able to provide connectivity to the mobile devices in the form of an IP private network with up to eight devices. Each PC-stick “sees” the IR/VLC dongle connected to the USB port as a network interface, in a manner similar to what happens to a normal device equipped with a WiFi card. On this network it is therefore possible to use the typical protocols of the TCP/IP protocol stack.

**Figure 3:**
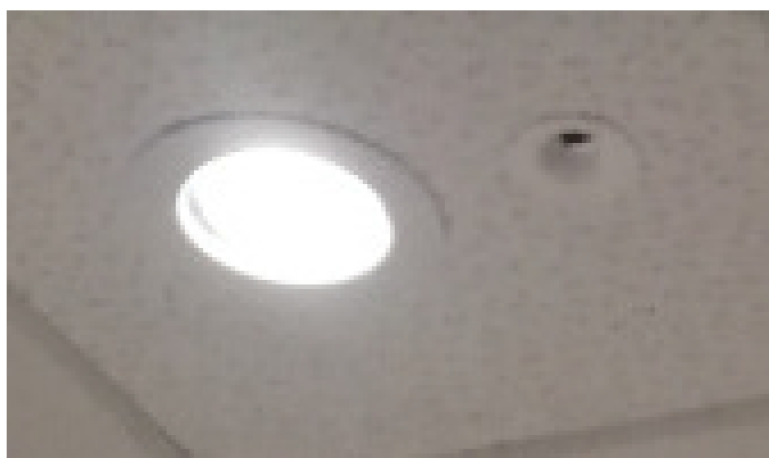
LED lamp and IR sensor mounted on the ceiling.

The APs, installed in the false ceiling, are composed by the main element, shown in Figure 3, mounted in the false ceiling with the IR reception sensor facing downwards, and by the LED lamp, shown in Figure 3 already plugged into the false ceiling. The lamp is a 20 W 4000 K Lucicup II by Lucibel, with a maximum luminous power of 1930 lm.

**Figure 4:**
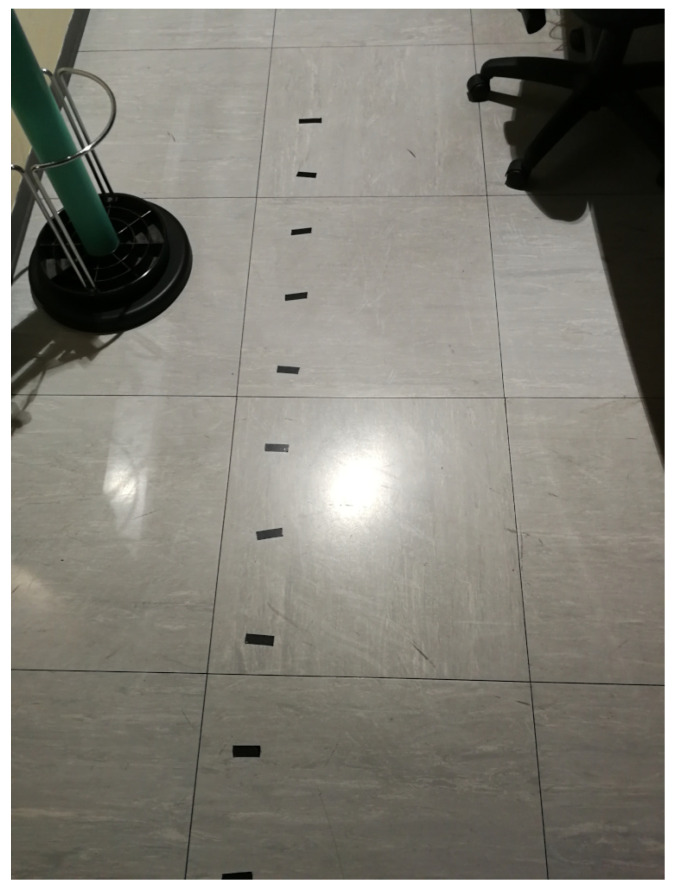
Floor notches.

**Figure 5:**
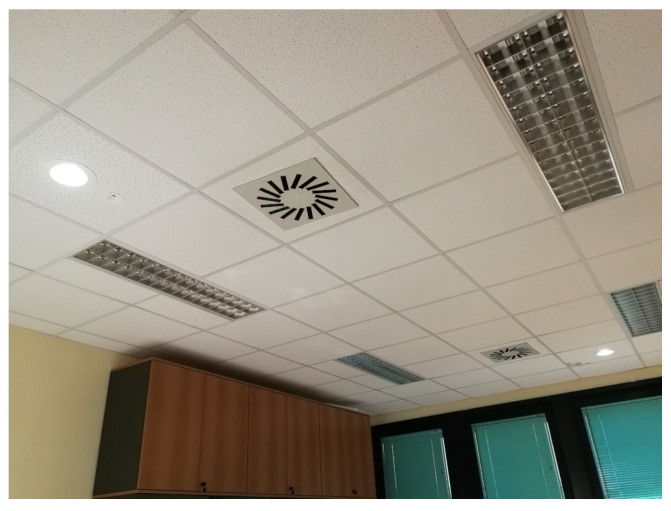
Ceiling: LED lamps, IR sensors, and neon lights.

On the floor, adhesive tape notches were placed, at 20 cm intervals, to carry out the measurements with the VLC dongle positioned at a variable distance from the vertical projection (nadir), on the floor, of the position of the lamp on the ceiling, as shown in Figure 4. This distance is also known as the “off-nadir distance”. Finally, Figure 5 shows an overview of the ceiling, in which the two lamps and IR sensors corresponding to the two APs are visible, along with four neon lights (in this case off) which are used, together with the shutters visible in the background, to modulate the background luminosity in one of the experiments carried out.

## 5. Performance Evaluation

We have carried out different tests to examine the throughput that is achieved in the communications in both directions:

- Downlink: from APs to mobile devices, using LED lamps, each connected to an AP, as transmitters in the visible light band, and the light receiver on the LiFi dongles as receivers.
- Uplink: from mobile devices to APs, using the IR transmitters on the LiFi dongles as transmitters and the IR sensors placed on the ceiling as receivers.

Based on information directly obtained by us from the producer of the LiFi equipment, PureLifi [53], the IR and visible light transmissions use OFMD at the physical layer and TDMA for handling multiple users.

Each experiment consists of the transmission of a data stream packaged according to the specifications of either of two protocols, TCP and UDP, in one of the two directions. The duration of the experiment is variable, ranging from 60 to 600 s, depending on the experiment. The achieved throughput is measured in Mbps. As already mentioned, to measure the achieved throughput, we used the iPerf speed test tool [52]. To evaluate the possible distortion effect of a specific network protocol in use, the measurements were performed with different standard protocols, TCP and UDP, both supported by the IP network protocol. However, we found that the difference of throughput in the use of the two protocols is relatively small. Each measurement is performed (with the exception of the first one) with the goal to highlight the performance of the system with respect to the variation of a system, environmental, or deployment-related parameter. In Table 1 we summarize the considered parameters. For each parameter, we list the default value(s), i.e., the value used in all the experiments in which a given parameter does not vary, and the value range considered in the experiment in which it is varied. Experiments are referred to using the respective figure number.

Before proceeding with the description of the experimental results in the next subsections, it is worth spending a few words on the order of magnitude of the performance we measured in terms of throughput: the nominal “raw” throughput of the LiFi transceivers used in our experiments is 43 Mbps in both downlink and uplink. Even taking into account the protocol overhead introduced by TCP and UDP, we will see that the measured performance are well behind this nominal value, as we reached peaks of ~32 Mbps in downlink and ~20 Mbps in uplink. This is the usual behavior of commercial devices. Moreover, our interest in this work is not to evaluate the performance in absolute terms, if not for their order or magnitude, which entail the capability (or not) to support specific applications. Rather, we are interested in the system reliability considering different operating conditions, i.e., to check if the system is able to guarantee relatively good performance, with respect to the peak value, even in cases when it operates in environmental and deployment conditions far from the most favorable ones, i.e., those that allow us to reach the peak data rates.

### 5.1. Single User Communication

In this experiment, a mobile device is positioned on a measurement table (at a 1-m height from the floor) directly under the LiFi AP.

**Figure 6:**
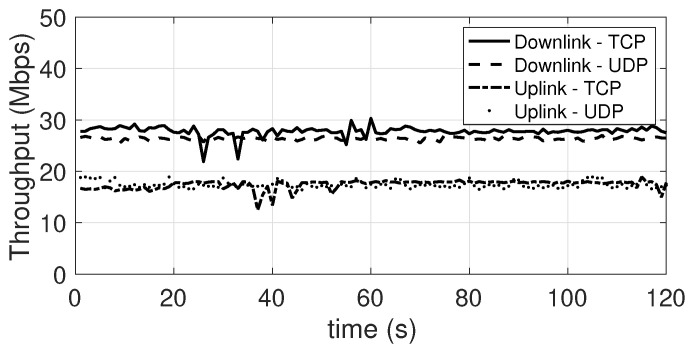
Single user performance. Downlink and Uplink with both TCP and UDP.

Figure 6 shows the throughput achieved in both directions and with both protocols. In downlink, the throughput is about 29 Mbps with the TCP protocol and about 27 Mbps with the UDP protocol. In Uplink, a throughput of ~17 Mbps is achieved with the TCP protocol and ~18 Mbps with the UDP protocol.

As a first result, it can be seen that the connections in both directions show a rather stable throughput, a characteristic that was found in most of the experiments performed, with the exception of the experiments in which the environmental or deployment conditions were varied during the experiment. The achieved throughput is adequate to support video streaming applications in both directions, as well as data flows from sensors for the acquisition of environmental or bio-medical parameters, which typically have a much lower throughput.

### 5.2. Concurrent Communication with Up to Six Mobile Devices

**Figure 7:**
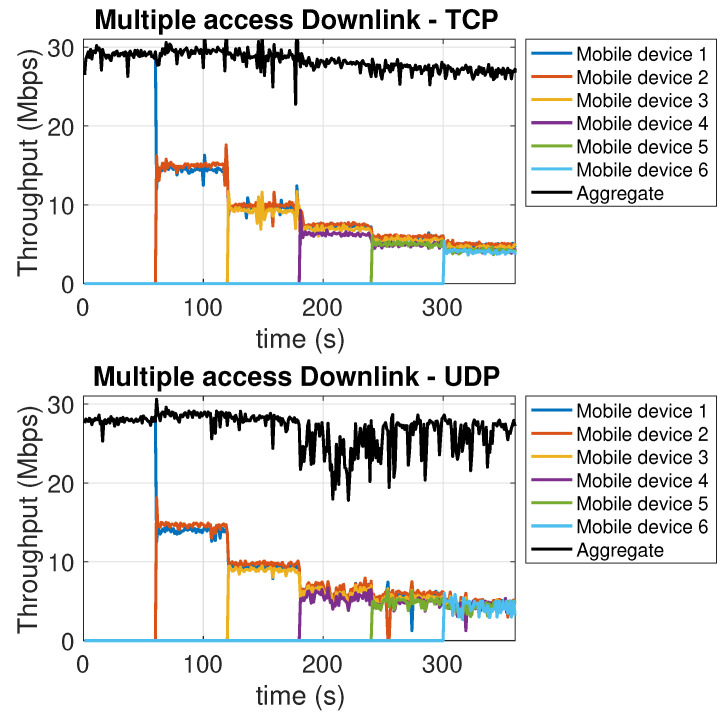
Multiple access *downlink* with TCP (top chart) and UDP (bottom chart).

**Figure 8:**
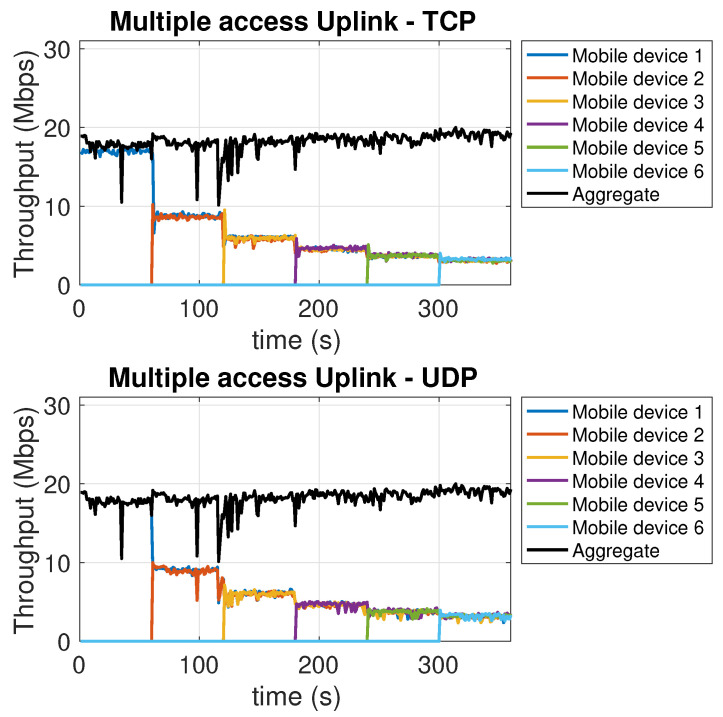
Multiple access *uplink* with TCP (top chart) and UDP (bottom chart).

Similarly to the above described experiment, in this experiment a number of communications up to a maximum of 6 are progressively added to pre-existing communications, to/from different mobile devices. From the downlink traces in Figure 7, we see a decrease in the throughput per user proportional to the reduced amount of bandwidth available for each user, but not exceeding this linear bandwidth decrease in a significant way. TCP and UDP, in this case, perform in a fairly similar way. For the uplink, Figure 8 shows a similar decrease, proportional to the reduction of the available per user bandwidth. A different behavior, between downlink and uplink, can be observed in terms of the aggregate throughput. For the downlink, both for TCP and UDP, most operations, including the flow control ones, are carried out by the single AP network interface. This may slow down the operations as more and more devices join. In the uplink, flow control and ACK reception and handling, carried out at the transmitter side, are now executed separately by the mobile devices interfaces. Therefore, the additional computational burden does not affect the aggregate throughput. In this case, in fact, the overall aggregate throughput shows a (small) increase for both TCP and UDP. Finally, we point out that the overall available bandwidth is always (re-)allocated to the users in a fair way, and this happens quickly. In fact, in both Figure 7 and Figure 8, as each new user comes in, the related plot almost superimposes to the preexisting ones, which, at the same time, present a downward step.

### 5.3. Performance Dependence on the LED Lamp Luminous Power

**Figure 9:**
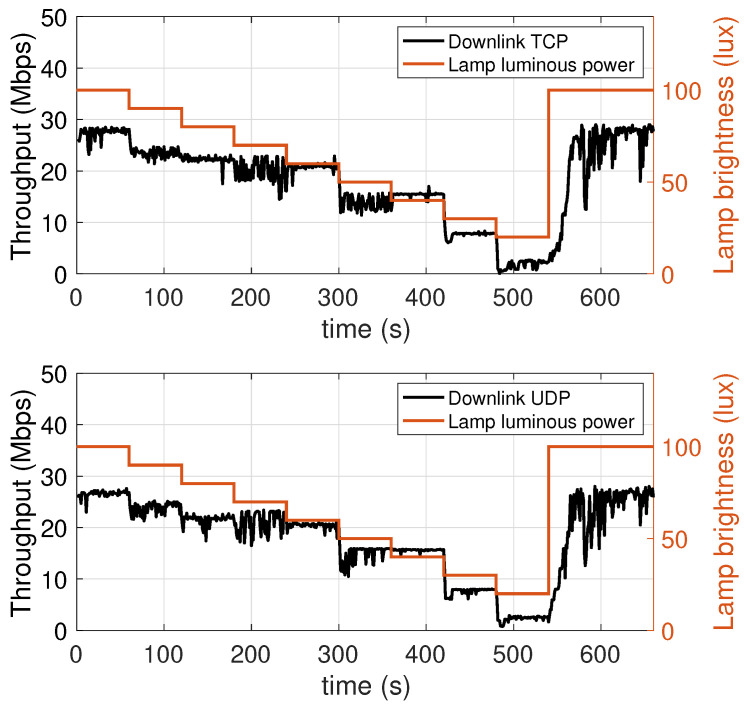
Downlink throughput with different lamp luminous power values. TCP (top chart) and UDP (bottom chart).

**Figure 10:**
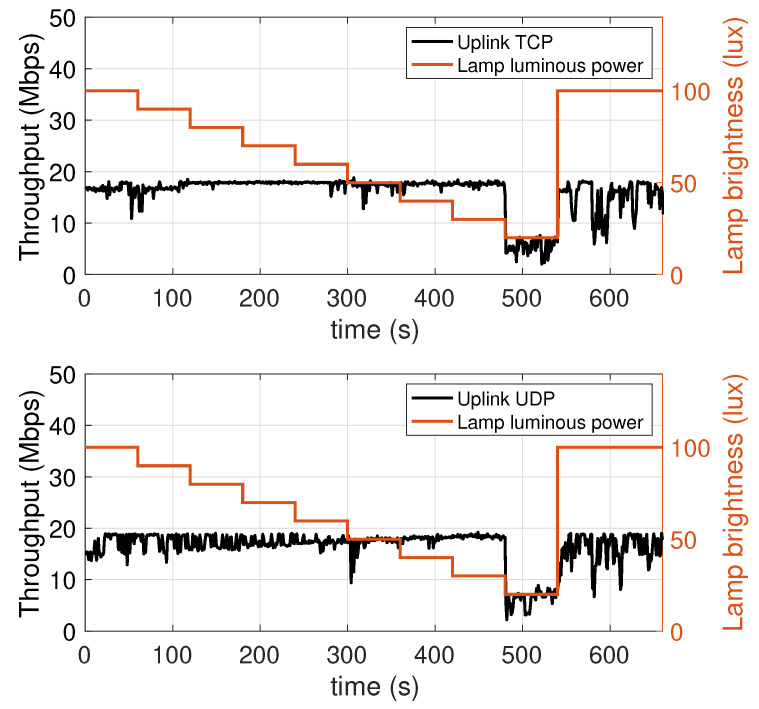
Uplink throughput with different lamp luminous power values. TCP (top chart) and UDP (bottom chart).

To measure the dependence of the performance on the luminous power of the LED lamp, we carried out transmissions during which the luminous power was progressively decreased. Specifically, starting from the maximum value of 1930 lm, every 60 s this was decreased by 10% of the maximum value (i.e, in steps of 193 lm), until a minimum of 386 lm (equal to 20% of the maximum luminous power), and then set back to the maximum value in the last interval. In Figure 9 (downlink) and Figure 10 (uplink) two scales appear in the vertical axes: the left axis reports the value of the achieved throughput, the right axis indicates the value of the lamp luminous power, measured in terms of percentage of the maximum value of 1930 lm. The red plot values, reproducing the staircase decrease of the lamp luminous power, map to the right axis.

Considering the downlink (Figure 9), it can be observed that the throughput depends on the lamp luminous power. Considering an initial throughput of 27.5 Mbps, we see that with the first decrease in luminous power there is a throughput drop of around 5 Mbps. In the following four intervals, corresponding to the luminous power values of 90%, 80%, 70%, and 60% of the maximum value, the throughput remains more stable, with a slight cumulative decrease of around 2.5 Mbps, ending up with a throughput of 21 Mbps at 300 s. The subsequent decrease in luminous power, from 60% to 50% of the maximum value, i.e., 965 lm, involves a new significant throughput drop of more than 5 Mbps. The throughput then remains at a decent level of 15 Mbps even at 40% of the maximum value. It then has a sharp drop of around 8 Mbps as the luminous power is diminished to 30%, and in the passage from 30% to 20% (386 lm) a further 5 Mbps drop can be seen, as the throughput reaches the minimum detected value of 2 Mbps. Finally, when the LED lamp is brought back to 100% of the maximum luminous power, the throughput increases again, but it takes about 20 s to complete this increase. This is likely due to the need for the network protocols to adapt their parameters to the new conditions. It is interesting to observe that with UDP, which is a connectionless and stateless protocol with no flow control mechanism and packet retransmissions, the throughput increase takes an amount of time similar to the TCP case, which is a connection-oriented protocol implementing such mechanisms. This suggests that most of this effect is due to the proprietary link layer protocols. This aspect, however, requires further investigation, which we reserve for our future work.

The conclusions that can be drawn are that, in the range between 40% (772 lm) and 100% (1930 lm), the system is able to guarantee considerable throughputs, between 15 and 30 Mbps. This stability can be used to optimize the luminous power level, and hence the electric power consumption, considering, for instance, the type of use which is being made of the lamp: if, at a given time, the lamp is being use for either communication or illumination purposes, or for both, this can make a difference in the selection of the optimal level. For low luminous power levels, 20% and 30%, the throughput is instead less than 8 Mbps. This should be kept in mind when considering nightly scenarios, or scenarios where weak or very weak lighting is required.

Considering the uplink (Figure 10), the behavior is completely different from what can be seen for the downlink. Particularly, the throughput is substantially invariant with respect to the luminous power of the LED lamp, at least as long as this is kept on values larger than or equal to 30% of the maximum value.

This behavior is expected, as the intensity of emission that, in the uplink case, is more directly related to the performance is that of the mobile transmitter (which operates in the infrared band). However, when the luminous power of the AP lamp falls below 30%, there still is a decrease in the uplink throughput. This happens because, to keep the uplink data flow active, the mobile device needs to periodically receive signalling packets (e.g, ACK messages) from the AP, which obviously transmits them using the LED lamp. If the communication quality of the transmission of these packets degrades, and thus some are lost, the mobile device is led to believe that some of the packets it has transmitted have not been received, thus resulting in a lower throughput.

### 5.4. Dependence on the Distance from to the Vertical Projection of the Lamp on the Floor

**Figure 11:**
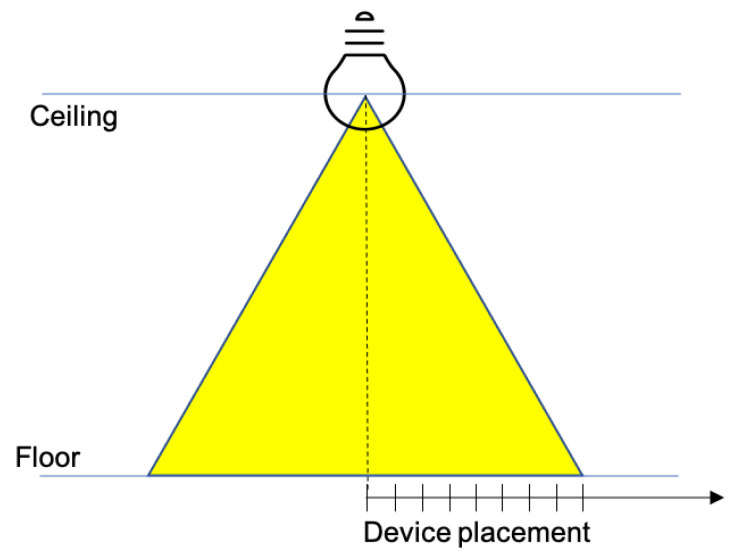
LED lamp coverage cone.

**Figure 12:**
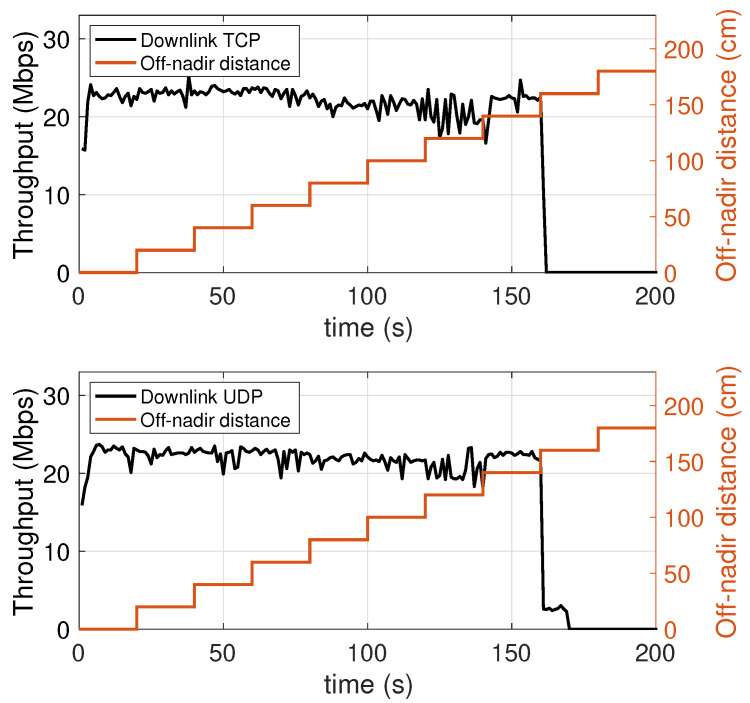
Downlink throughput with varying off-nadir distance of the mobile device. TCP (top chart) and UDP (bottom chart).

**Figure 13:**
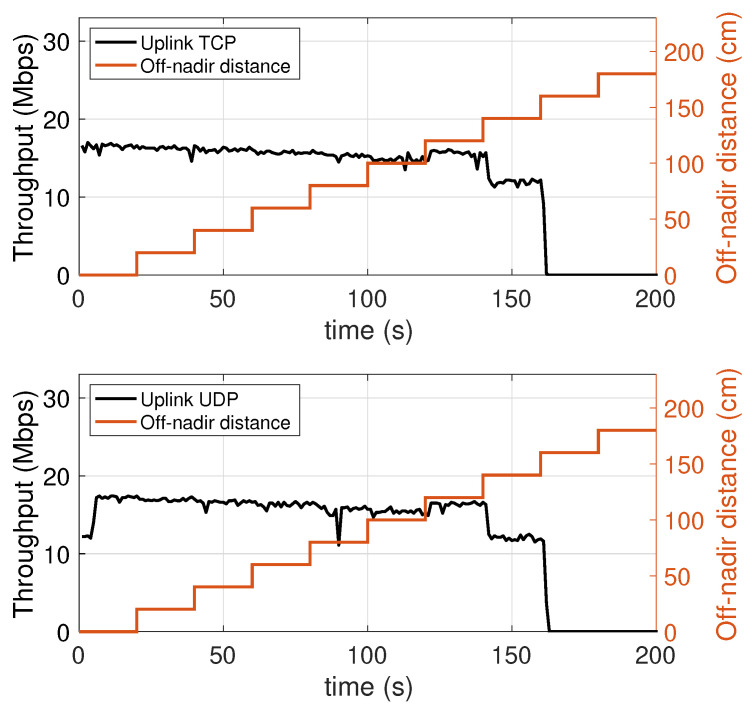
Uplink throughput with varying off-nadir distance of the mobile device. TCP (top chart) and UDP (bottom chart).

In this experiment, the mobile device is placed on the floor of the test environment, and moved along a straight line away from the projection of the lamp on the floor (the nadir point), until it exits the attocell coverage cone, as shown in Figure 11 (see also Figure 4). During the experiment, the off-nadir distance is varied according to the red plot appearing in Figure 12 and Figure 13. The values of this distance are shown on the right vertical axis, from 0 to 180 cm. It is found that the performance, both in the downlink (Figure 12) and in the uplink (Figure 13), undergo a relatively small degradation as the device is moved away from the center of the the coverage cone projection, approaching its edge. A slightly more sensitive decay occurs in the uplink near the cell edge, in the passage from 120 cm (at which an uplink throughput of about 14 Mbps is still obtained) to 140 cm, at which the uplink throughput is around 12 Mbps. Obviously, the throughput decays to zero when the device exits the direct illumination cone of the lamp. The radius of the coverage region at the floor level is therefore around 150 cm, for a diameter of 3 m.

### 5.5. Dependence on the Orientation of the Mobile Device with Respect to the Connection with the Access Point

**Figure 14:**
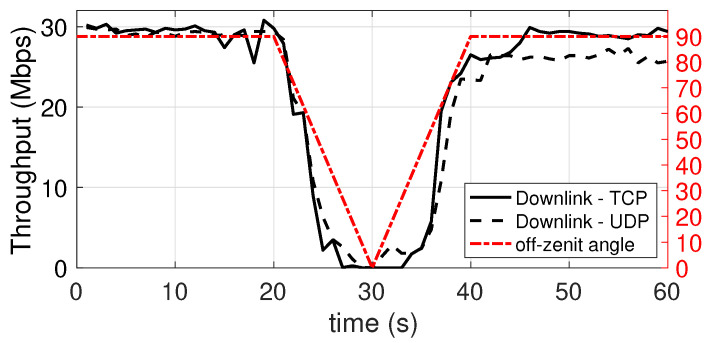
Downlink throughput with different orientation angle of the mobile device. TCP and UDP.

**Figure 15:**
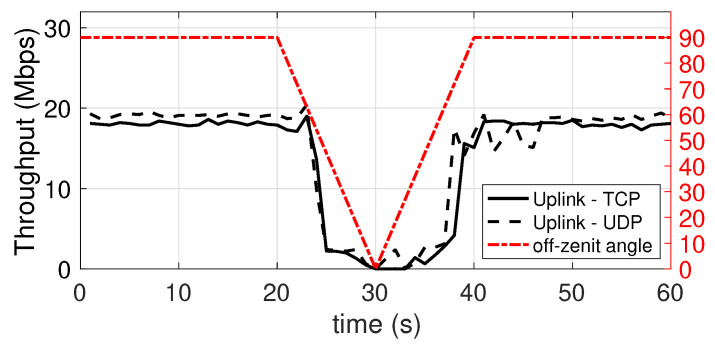
Uplink throughput with different orientation angle of the mobile device. TCP and UDP.

In this experiment, the achieved throughput, both in the downlink and in the uplink, is evaluated as the orientation of the mobile device varies. The initial orientation is 90°, i.e., the device is in an horizontal position, right under the lamp on the ceiling, with the LED and IR sensor directly point upwards, to the lamp. The device stays in this position for 20 s. In the next 10 s the device is rotated (at a constant speed) by 90 degrees around a horizontal axis, until it points to the side wall, i.e., 0°. Subsequently, again taking 10 s, it is returned to the original position, and it is kept in this position until the end of the measurement. The plots shown in Figure 14 and Figure 15 show the performance in downlink and uplink, respectively. The red hash-dot curve, whose values are mapped to the right vertical axis, represents the angle of orientation (with 0° representing the device LED and IR sensor pointing to the side wall). It can be seen that the performance undergoes significant degradation, similar to a sinusoidal trend. However, in both cases, up to a misalignment of around 45°, the connection substantially provides an acceptable throughput, retaining at least 50% of the peak speed.

### 5.6. Dependence on the Height at Which the Mobile Device Is Placed

**Figure 16:**
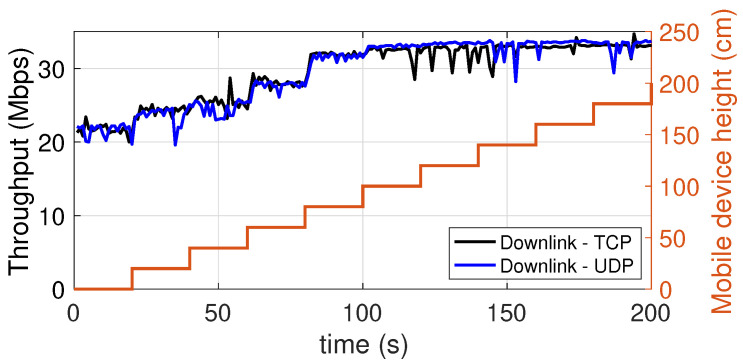
Downlink throughput with the mobile device placed at different heights. TCP and UDP.

**Figure 17:**
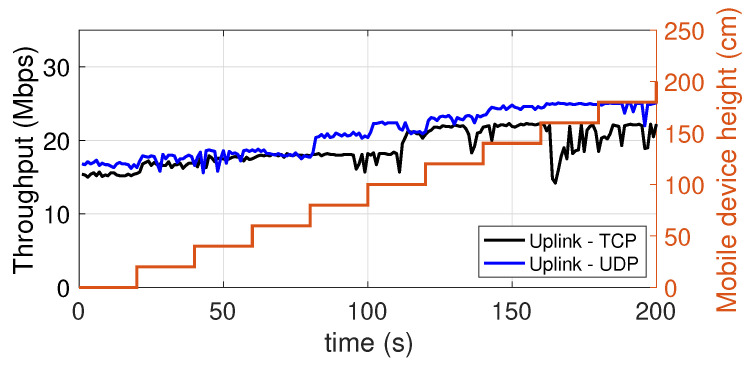
Uplink throughput with the mobile device placed at different heights. TCP and UDP.

In this experiment, we evaluated the dependence of the throughput on the height at which the mobile device is placed. In Figure 16 (downlink) and Figure 17 (uplink) there are two vertical axes. The axis on the right represents the height at which the device was placed during a measurement period of 120 s. In particular, starting from the floor level, the height is increased in steps of 20 cm every 20 s, up to a maximum of 180 cm. As it can be seen, the results are excellent. In fact, in the downlink, already at the floor level there is a throughput equal to 22 Mbps, or 66% of the maximum value of 33 Mbps, which is reached at a height of just 90 cm. In the uplink, the situation is even better in terms of height sensitivity, as at floor level the throughput of 15–17 Mbps oscillates between 70% and 75% of the maximum value of 20–24 Mbps (depending on the protocol used).

### 5.7. Effect of the Transition from the Coverage Area of One Access Point to That of the Other Access Point (Handover)

**Figure 18:**
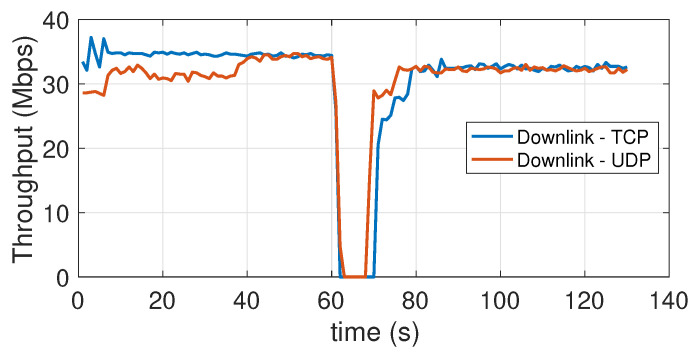
Downlink throughput during an handover across close-by attocells. TCP and UDP.

**Figure 19:**
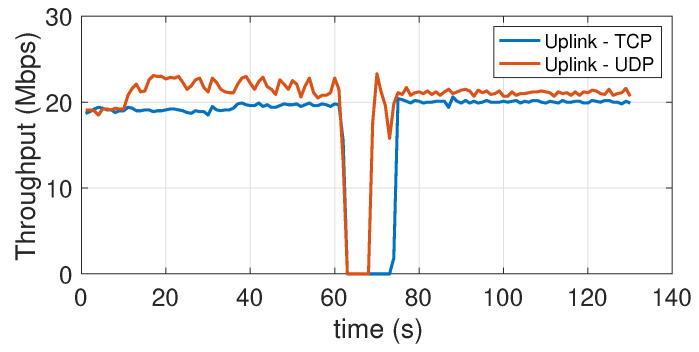
Uplink throughput during an handover across close-by attocells. TCP and UDP.

The aim of this experiment is to detect the reactivity of the system with respect to the mobility of a device across two close-by cells. In the testbed scenario, the coverage areas are not exactly adjacent, as the APs have been placed so as to leave a shadow area of about one meter (at floor level) between their coverage regions. To ensure a more stable angle orientation during the experiment (to avoid contamination of the results from the orientation effect, already described above) we have conducted it with the USB dongle connected to a laptop. The use of a laptop, i.e., a device with a more powerful CPU, could also justify the slightly larger value of the peak throughput in Figure 18 with respect to the peak value visible in Figure 16. The laptop, kept at an height of 150 cm, is moved relatively quickly, within a couple seconds, from the coverage region of one AP to that of the other. The moment in which this occurs is clearly visible in the throughput traces of Figure 18 for the downlink, and Figure 19 for the uplink. From the downlink traces, we can see how the response time of the device to re-establish the connection (with the new AP) is about 10 s for the TCP protocol and about 5 s for the UDP protocol.

In the uplink the situation is reversed, as with TCP re-establishing the connection takes about 5 s, while with UDP it takes 10 s. In general, the behavior of the system, from this point of view, is not particularly brilliant, as these connection recovery intervals are rather large, even taking into account the time taken (2~3 s) to physically move the device from one area to the other. However, it should be noted that this is one of those aspects with respect to which substantial improvements can be expected in the coming years, as highlighted by specific works in the literature, for instance [50,51].

### 5.8. Dependence on the Background Light Conditions

**Figure 20:**
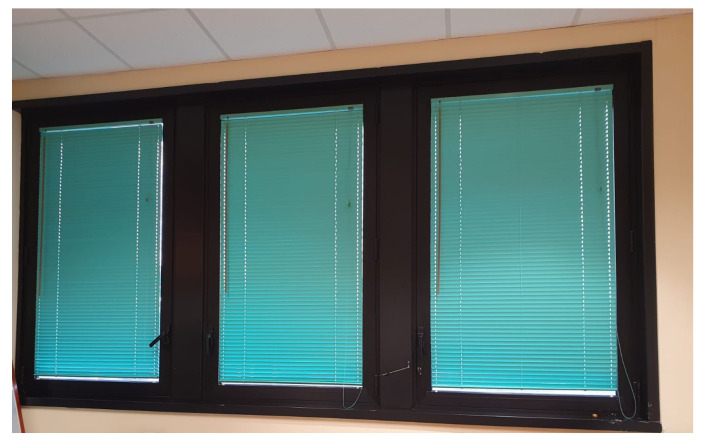
Windows shutters.

In this experiment, the effect of the intensity of the background light of the test environment is studied. The experiment considers two levels of the LED lamp luminous power: 100% of the maximum luminous power, and 40%, which provides moderate lighting conditions. Different operating conditions were considered. Specifically:

- Two conditions of the neon in the room have been considered: neon all on or neon all off.
- For each scenario resulting from the four possible combinations of neon lighting and LED lamp luminous power, four open/close combination of the three roller shutters in the room were considered, indicated below with the letters A, B, C, D. The difference between one condition and another lies in the number of open or closed shutters. When a shutter is closed, it is intended that the flaps are oriented so as to let the minimum amount of external light penetrate in the room. Figure 20 shows the shutters used to impose the different lighting conditions.

Table 2 summarizes the operating conditions considered for this experiment, the sub-tables present in the four quadrants show the possible configurations A, B, C, D of the shutters (numbered with 1, 2, 3), in which the cells in black represent the closing state of a shutter, while the cells in white indicate the opening state, that is the state in which the shutter is completely raised.

**Figure 21:**
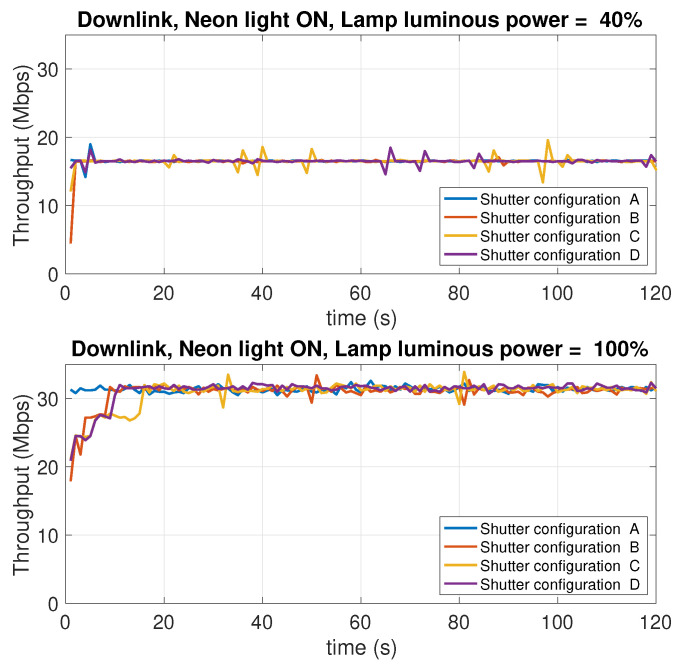
Downlink throughput with different shutter configurations (see Table 1). Neon lights ON. LED lamp luminous power: 40% (top charts) and 100% (bottom charts) of the maximum luminous power of 1930 lm.

**Figure 22:**
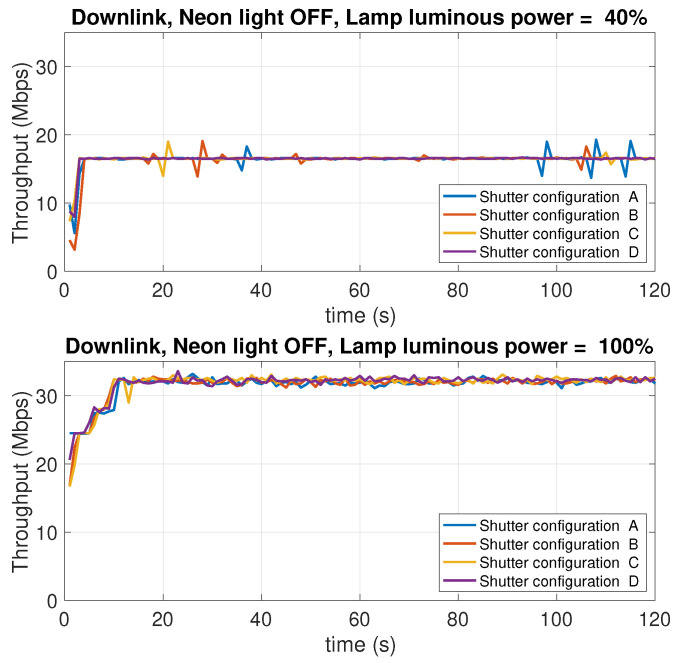
Downlink throughput with different shutter configurations (see Table 1). Neon lights OFF. LED lamp luminous power: 40% (top charts) and 100% (bottom charts) of the maximum luminous power of 1930 lm.

**Figure 23:**
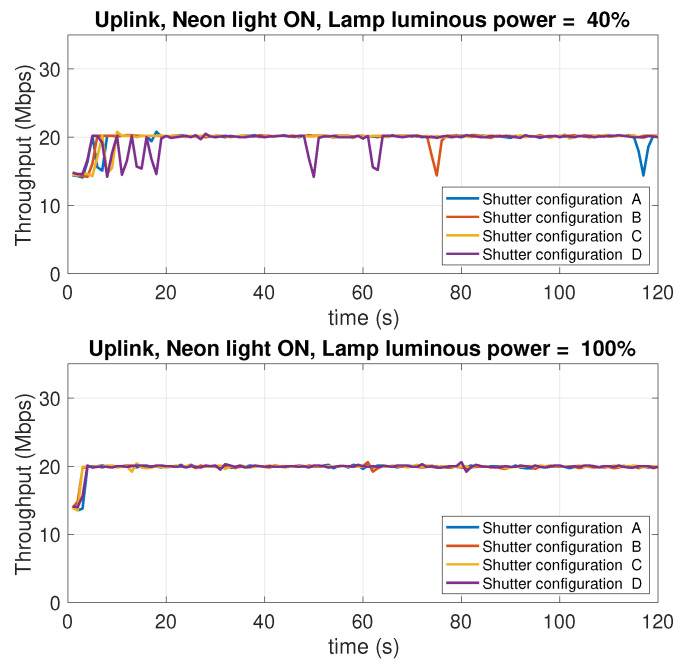
Uplink throughput with different shutter configurations (see Table 1). Neon lights ON. LED lamp luminous power: 40% (top charts) and 100% (bottom charts) of the maximum value of 1930 lm.

**Figure 24:**
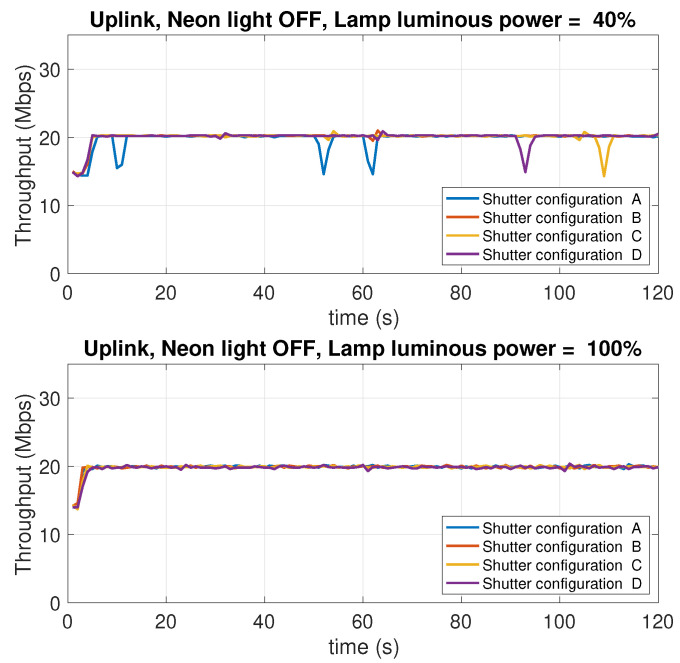
Uplink throughput with different shutter configurations (see Table 1). Neon lights OFF. LED lamp luminous power: 40% (top charts) and 100% (bottom charts) of the maximum value of 1930 lm.

There is, hence, a total of 16 measurement scenarios. For each scenario, the downlink and uplink throughput is evaluated. In this case, only the TCP protocol was used. Figure 21, Figure 22, Figure 23 and Figure 24 report the results of the experiments.

Observing the curves in the four figures, the following conclusions can be drawn:

- As the luminous power of the LED lamp varies between 100% of the maximum value (1930 lm) and 40% (772 lm) the downlink performance (from the AP to the mobile device) undergoes a decrease from 30 Mbps to 17 Mbps. On the contrary, the uplink performance (from the mobile device to the AP) remain unchanged. This is evident considering the performance traces at the top of each figure (for the downlink), and those at the bottom (for the uplink).
- As the neon lighting status changes, the performance do not change, both in uplink and downlink.
- As the number of closed or open shutters varies, the performance do not change, both in uplink and downlink.

In conclusion, it can be said that, in the scenario considered in our experiments, both uplink and downlink performance are not affected by background lighting in any way. The performance in downlink is only affected by the luminous power radiated by the LED lamp. Finally, the uplink performance is also independent of this factor. Although there are a few studies in the literature that have investigated, or taken into account, the effect of background light, most of them deal with harsh environments, like outdoor vehicular environments [54], where the sun light can hit the photodiodes either directly or through reflections on the cars’ surfaces, or industrial environments [55], where dust in the air can compromise the transmissions as it scatters the background light causing flickering noise. From this perspective, our scenario is clearly more favorable. The take home message of our results is that, in an indoor scenario with no direct sunlight impinging on the photodiodes, and a relatively clean air, commercial systems (at least the one we have considered) guarantee background-light independent performance.

## 6. Conclusions

In this study, we have presented the results of a series of tests performed by us to investigate the dependence of the performance of an IR/VLC based LAN from different environmental and deployment factors in an indoor environment. From the performance evaluation carried out in the previous subsections, we can conclude that the connections established both in uplink and downlink are rather robust and stable with respect to almost all the operational and environmental parameters considered. the throughputs obtained using devices available on the market are already adequate to support many applications that can be envisaged for indoor office and domestic scenarios. However, taking into account that this is a technology currently subject to intense research by the scientific community, it has to be expected that commercial products will increase their performance in a matter of very few years, thus being able to support more application uses and/or more devices.

## Figures and Tables

**Table 1:** Parameters setting.

Parameter	Experiment Reference	Fixed Value or Value Range
Number of devices	All the figures *but* Figures 7 and 8	1
	Figures 7 and 8	1–6
Luminous power	All the figures *but* Figures 9 and 10	1930 lm (or 100% Max power)
	Figures 9 and 10	[386–1930] (lm) (or [20–100%] of Max power)
Off-nadir distance	All the figures *but* Figures 12 and 13	0 cm
	Figures 12 and 13	[0–180] (cm)
Device height	All the figures *but* Figures 12, 13 and 16–19	90 cm
	Figures 12 and 13	0 cm
	Figures 16 and 17	[0–180] (cm)
	Figures 18 and 19	150 cm
Device pointing angle	All the figures *but* Figures 14 and 15	90°
	Figures 14 and 15	[0–90°] (degrees)
Room window shutters	All the figures *but* Figures 21–24	All open
	Figures 21–24	See Table 2
Room neon lights	All the figures *but* Figures 21–24	All ON
	Figures 21–24	See Table 2

**Table 2:** Background illumination combinations.

	Lamp Luminous Power	Luminous Power = 772 lm (40% Maximum Value)	Luminous Power = 1930 lm (100% Maximum Value)
Neon State			
Neon ON		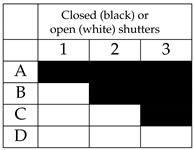	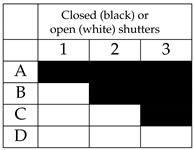
Neon OFF		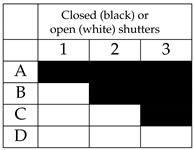	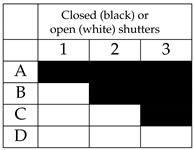

## Data Availability

The data presented in this study are available on request from the corresponding author.
